# A Laboratory Medicine Best Practices Systematic Review and Meta-analysis of Nucleic Acid Amplification Tests (NAATs) and Algorithms Including NAATs for the Diagnosis of *Clostridioides* (*Clostridium*) *difficile* in Adults

**DOI:** 10.1128/CMR.00032-18

**Published:** 2019-05-29

**Authors:** Colleen S. Kraft, J. Scott Parrott, Nancy E. Cornish, Matthew L. Rubinstein, Alice S. Weissfeld, Peggy McNult, Irving Nachamkin, Romney M. Humphries, Thomas J. Kirn, Jennifer Dien Bard, Joseph D. Lutgring, Jonathan C. Gullett, Cassiana E. Bittencourt, Susan Benson, April M. Bobenchik, Robert L. Sautter, Vickie Baselski, Michel C. Atlas, Elizabeth M. Marlowe, Nancy S. Miller, Monika Fischer, Sandra S. Richter, Peter Gilligan, James W. Snyder

**Affiliations:** a Emory University School of Medicine, Atlanta, Georgia, USA; b Department of Interdisciplinary Studies, School of Health Professions, Rutgers University, Newark, New Jersey, USA; c Department of Epidemiology, School of Public Health, Rutgers University, Piscataway, New Jersey, USA; d Centers for Disease Control and Prevention, Atlanta, Georgia, USA; e Microbiology Specialists Incorporated, Houston, Texas, USA; f American Society for Microbiology, Washington, DC, USA; g Perelman School of Medicine, University of Pennsylvania, Philadelphia, Pennsylvania, USA; h Accelerate Diagnostics, Tucson, Arizona, USA; i Children’s Hospital Los Angeles, Los Angeles, California, USA; j Keck School of Medicine, University of Southern California, Los Angeles, California, USA; k Kaiser Permanente (Southern California Permanente Medical Group) Regional Reference Laboratories, Greater Los Angeles, Los Angeles, California, USA; l University of California—Irvine, Orange, California, USA; m PathWest Laboratory Medicine, Perth, Western Australia, Australia; n University of Western Australia, Perth, Western Australia, Australia; o Rhode Island Hospital/Lifespan Academic Medical Center, Warren Alpert Medical School of Brown University, Providence, Rhode Island, USA; p RL Sautter Consulting LLC, Lancaster, South Carolina, USA; q University of Tennessee Health Science Center, Memphis, Tennessee, USA; r Kornhauser Health Sciences Library, University of Louisville, Louisville, Kentucky, USA; s Roche Molecular Systems, Pleasanton, California, USA; t Boston Medical Center, Boston, Massachusetts, USA; u Boston University School of Medicine, Boston, Massachusetts, USA; v Indiana University, Indianapolis, Indiana, USA; w Cleveland Clinic, Cleveland, Ohio, USA; x University of North Carolina School of Medicine, Chapel Hill, North Carolina, USA

**Keywords:** *C. difficile* infection, diagnostic accuracy, laboratory diagnosis, meta-analysis, systematic review

## Abstract

The evidence base for the optimal laboratory diagnosis of *Clostridioides* (*Clostridium*) *difficile* in adults is currently unresolved due to the uncertain performance characteristics and various combinations of tests. This systematic review evaluates the diagnostic accuracy of laboratory testing algorithms that include nucleic acid amplification tests (NAATs) to detect the presence of C. difficile. The systematic review and meta-analysis included eligible studies (those that had PICO [population, intervention, comparison, outcome] elements) that assessed the diagnostic accuracy of NAAT alone or following glutamate dehydrogenase (GDH) enzyme immunoassays (EIAs) or GDH EIAs plus C. difficile toxin EIAs (toxin). The diagnostic yield of NAAT for repeat testing after an initial negative result was also assessed. Two hundred thirty-eight studies met inclusion criteria. Seventy-two of these studies had sufficient data for meta-analysis. The strength of evidence ranged from high to insufficient. The uses of NAAT only, GDH-positive EIA followed by NAAT, and GDH-positive/toxin-negative EIA followed by NAAT are all recommended as American Society for Microbiology (ASM) best practices for the detection of the C. difficile toxin gene or organism. Meta-analysis of published evidence supports the use of testing algorithms that use NAAT alone or in combination with GDH or GDH plus toxin EIA to detect the presence of C. difficile in adults. There is insufficient evidence to recommend against repeat testing of the sample using NAAT after an initial negative result due to a lack of evidence of harm (i.e., financial, length of stay, or delay of treatment) as specified by the Laboratory Medicine Best Practices (LMBP) systematic review method in making such an assessment. Findings from this systematic review provide clarity to diagnostic testing strategies and highlight gaps, such as low numbers of GDH/toxin/PCR studies, in existing evidence on diagnostic performance, which can be used to guide future clinical research studies.

## Abstract (precis)

The evidence base for the optimal laboratory diagnosis of *Clostridioides* (*Clostridium*) *difficile* in adults is currently unresolved due to the uncertain performance characteristics and various combinations of tests. This systematic review evaluates the diagnostic accuracy of laboratory testing algorithms that include nucleic acid amplification tests (NAATs) to detect the presence of C. difficile.

## INTRODUCTION

*Clostridioides* (*Clostridium*) *difficile* infection (CDI) is the leading cause of health care-associated infections in the United States (1, 2). It accounts for 15% to 25% of health care-associated diarrhea cases in all health care settings, with 453,000 documented cases of CDI and 29,000 deaths in the United States in 2015 (3). Acquisition of C. difficile as a health care-associated infection (HAI) is associated with increased morbidity and mortality. This adds a significant burden to the health care system by increasing the length of hospital stay and readmission rates, with significant financial implications. The cost of hospital-associated CDI ranges from $10,000 to $20,000 per case (4–7) and $500 million to $1.5 billion per year nationally (1, 4, 5, 8–10).

Accurate diagnosis of CDI is critical for effective patient management and implementation of infection control measures to prevent transmission (11). The diagnosis of CDI requires the combination of appropriate test ordering and accurate laboratory testing to differentiate CDI from non-CDI diarrheal cases, including non-CDI diarrhea in a C. difficile-colonized patient (8). Accurate diagnosis of CDI is critical for appropriate patient management and reduction of harms that may arise from diagnostic error (12) and is critical for implementation of infection control measures to prevent transmission (11). Consequently, among patients presenting with diarrhea, there is significant potential for underdiagnosis or overdiagnosis as can arise from incorrect diagnostic workups (13).

### Quality Gap: Factors Associated with the Laboratory Diagnosis of C. difficile

Best practices for laboratory diagnosis of CDI remain controversial (14). Current laboratory practice is not standardized, with wide variation in test methods and diagnostic algorithms. Several laboratory assays are available to support CDI diagnosis in combination with clinical presentation. These include toxigenic culture (TC); the cell cytotoxicity neutralization assay (CCNA); enzyme immunoassays (EIAs) and immunochromatographic assays for the detection of glutamate dehydrogenase (GDH), toxin A or B, or both toxins; and, within the last 10 years, nucleic acid amplification tests (NAATs). Currently, two tests, TC and the CCNA, serve as reference methods for the diagnosis of C. difficile infection (15). The principle of the TC test is to detect strains of C. difficile that produce a toxin(s) following culture on an appropriate medium. CCNA detects fecal protein toxins contained within the stool and is often referred to as fecal toxin detection (16). Unfortunately, both tests are slow and labor-intensive.

Commercially available NAATs for C. difficile detection include those based on PCR or loop-mediated or helicase-dependent isothermal amplification (17–20). The performance of NAATs and non-NAAT tests is commonly assessed using diagnostic accuracy measures for the presence of the organism (e.g., diagnostic sensitivity, diagnostic specificity, positive predictive value [PPV], and negative predictive value [NPV]). However, these measures may not directly link to the clinical definition of CDI or clinical outcomes, and some measures (e.g., PPV and NPV) are dependent on disease prevalence in the patient population being tested (8, 17, 19, 20). Finally, in addition to diagnostic sensitivity and specificity, other factors influence the choice of testing strategy, such as cost and turnaround time.

The diagnostic accuracies of current commercially available assays (GDH EIAs, toxin A/B EIAs, and NAATs) are based on comparison with one or both of the currently accepted reference methods (TC and CCNA) for the detection of toxigenic C. difficile, and these comparisons are generally made to inform potential replacement of these reference methods. Although a definitive reference “gold standard” is lacking, both TC and CCNA are regarded as acceptable reference methods (15). However, some view the gold standard to be TC of a stool specimen combined with colonic histopathology of pseudomembranous colitis in patients with symptoms, but it is known that there is a spectrum of disease wherein not all patients with C. difficile infection have pseudomembranes (21). Finally, less frequently, colonoscopic or histopathologic findings demonstrating pseudomembranous colitis can be used in diagnostic workups to increase the diagnostic specificity for CDI diagnosis (14).

In contrasting the two reference methods (TC and CCNA), TC, while infrequently performed in clinical laboratories, is regarded as being more analytically sensitive than CCNA for detecting C. difficile in fecal specimens but may have lower diagnostic specificity (and, therefore, a greater likelihood of false-positive [FP] test results). CCNA has been shown to have high diagnostic sensitivity, ranging from 80 to 100%. In addition, CCNA has high diagnostic specificity and positive predictive values as well as having greater clinical utility based upon clinical outcomes (22–26). Furthermore, each reference method differs by the target detected: TC detects the presence of C. difficile strains that produce toxins A and/or B *in vitro* to confirm a toxigenic strain, whereas CCNA detects the presence of free toxin A or B in clinical specimens. Given these contrasting characteristics, there is potential for diagnostic discrepancy between the reference standards. Therefore, observed diagnostic performance may vary according to which reference standard is used.

Given the variety of test methods and diagnostic algorithms, there is disagreement in the laboratory community on whether best practices for the diagnosis of CDI consist of NAAT only or algorithmic testing that includes NAAT (GDH EIA followed by NAAT [GDH/NAAT] or GDH and toxin EIAs followed by NAAT [GDH/toxin/NAAT]) (20). At the initiation of these guidelines, this was the clinical quandary facing individuals who decide on a C. difficile testing strategy for their health care system, particularly as there is limited high-quality evidence to support which diagnostic testing strategy best supports the laboratory diagnosis of CDI (8, 22). Additionally, it remains to be determined if the potential differences in the accuracy of NAAT only or an algorithmic strategy would impact patient management or patient outcomes (27). There are few studies that encompass the nuances of laboratory CDI diagnosis as it occurs in the clinical context, for example, that evaluate the effect of preanalytic testing considerations on outcomes, to include clinical outcomes. This limitation is evident from the recent Infectious Diseases Society of America (IDSA)/Society for Healthcare Epidemiology of America (SHEA) systematic review, which included only studies that encompassed C. difficile testing within its clinical context, including preanalytic and postanalytic aspects (11).

Given these practice issues, and related diagnostic quality and patient safety concerns, the goal of this systematic review was to determine which laboratory testing strategies, with the inclusion of NAAT, had the best diagnostic accuracy for CDI. While it is clear that laboratory testing alone without taking into consideration the entire clinical picture is not appropriate for the diagnosis of CDI, the available literature has limited evidence linking laboratory diagnosis with clinical outcomes. Therefore, the questions for this systematic review were refined to be based only on the intermediate outcome of diagnostic accuracy for detecting the presence of the C. difficile organism or toxin. Although the reference standard in these studies defines what is meant by the target condition, this systematic review compares the diagnostic accuracies of these tests, including GDH detection by EIA, toxin detection by EIA, and NAAT, to those of CCNA and TC. It has been clear that preanalytical factors are crucial for NAAT specifically, and many of the studies did not include a preanalytical component, which limits whether this review can answer the question, Does this patient have C. difficile infection?

The questions that guided this systematic review were the following: (i) What is the diagnostic accuracy of NAAT only versus either TC or CCNA for detection of the C. difficile toxin gene?, (ii) What is the diagnostic accuracy of a GDH-positive EIA followed by NAAT versus either TC or CCNA for detection of the C. difficile organism/toxin gene?, (iii) What is the diagnostic accuracy of a GDH-positive/toxin-negative EIA followed by NAAT versus either TC or CCNA for detection of the C. difficile organism/toxin/toxin gene?, and (iv) What is the increased diagnostic yield of repeat testing using NAAT after an initial negative result for C. difficile detection of the toxin gene?

The goals of analysis based on these questions were specifically to evaluate the effectiveness of the following: (i) the diagnostic accuracies of NAAT-only and algorithmic (“two-step” or “three-step”) testing strategies, including detection of toxin or GDH in addition to NAAT, and (ii) the diagnostic yield of repeat testing after an initial negative NAAT result. The evidence supporting these two important issues was evaluated by applying the Centers for Disease Control and Prevention (CDC) Laboratory Medicine Best Practices (LMBP) Initiative’s systematic review method for translating results into evidence-based recommendations (28). The method has recently been used to evaluate practices for improving blood culture contamination (29), blood sample hemolysis (30), urine culture sample quality (31), timeliness of providing targeted therapy for bloodstream infections (32), and laboratory test utilization (33), in addition to others, and can be found at the CDC LMBP website (https://www.cdc.gov/labbestpractices/our-findings.html).

## METHODS

This systematic review was guided by the CDC Division of Laboratory Systems (DLS) LMBP A-6 cycle, a previously validated evidence review and evaluation method for quality improvement in laboratory medicine that is reported in detail elsewhere (28). For additional resources, see www.cdc.gov/labbestpractices/index.html and https://www.cdc.gov/library/researchguides/sytemsaticreviews.html. The A-6 cycle was designed to assess the results of studies of practice effectiveness to derive evidence-based practice recommendations.

The systematic review was conducted by a review coordinator, a technical coordinator, a statistician with expertise in quantitative evidence analysis, and volunteer faculty (referred to as the expert panel), who were trained to apply CDC LMBP methods. The team worked with the independent, multidisciplinary LMBP Work Group consisting of 13 invited members with broad expertise in laboratory medicine, clinical practice, health services research, and health policy as well as one *ex officio* representative from the Centers for Medicare and Medicaid Services. They provided simultaneous, independent feedback on the analysis of the evidence base and the resultant American Society for Microbiology (ASM) practice recommendations. Appendix SA in the supplemental material lists the LMBP Work Group members and expert panel members and further describes their roles. The ASM Professional Practice Committee vetted the conflicts of interest for the volunteers who formed the expert panel. This systematic review and meta-analysis is intended to be updated every 5 years or as new data emerge that would change the results of this meta-analysis.

### Ask: Review Questions and Analytic Framework

**FIG 1:**
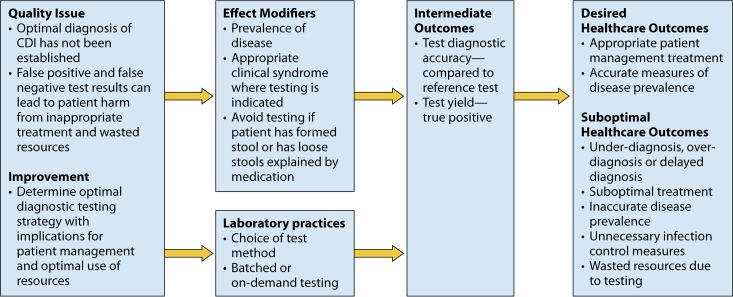
Analytic framework to address the question, How effective are NAAT practices for diagnosing patients suspected of having Clostridium difficile infection? Health care outcomes were not able to be considered in this systematic review; only intermediate outcomes could be assessed. On-demand versus batched testing could not be assessed because they were not listed in the literature.

The overarching question addressed through this systematic review is, How effective are NAAT practices for diagnosing patients suspected of having Clostridium difficile infection? This review question was developed in the context of the analytic framework depicted in Fig. 1.

The “PICO” (population, intervention, comparison, outcome) elements, which directly inform study eligibility criteria, are

- Population: any patients suspected of having a C. difficile infection, although pediatric populations (<18 years old) were excluded from analysis
- Intervention (index test): NAAT only or diagnostic algorithms containing at least one NAAT (e.g., two-step or three-step algorithms)
- Comparison (reference method): TC and/or CCNA
- Outcomes: diagnostic accuracy (sensitivity, specificity, positive and negative likelihood ratios [LRs], and diagnostic odds ratio) of C. difficile testing strategies.

Preanalytic data, such as history of antibiotic use or prior hospitalization, >3 unformed bowel movements within 24 h, age of the patient, residence in long-term-care facilities, and others, are often used by health care providers when deciding if a patient should be tested for the presence of toxigenic C. difficile. These preanalytic data were often not included in the diagnostic accuracy comparison studies (or, if included, were not reported within the study publication), representing a study (or study reporting) limitation that should be addressed in future studies before moving forward in clinical comparison studies. See Appendix SB in the supplemental material for a suggested data collection form designed for use in future studies.

### Acquire: Literature Search and Request for Unpublished Studies

The literature search strategy for eligible studies was based on input from the expert panel, a research librarian at the University of Louisville School of Medicine library in Louisville, KY, and a CDC medical librarian consultant. A search of three electronic bibliographic databases (PubMed, SCOPUS, and Embase) for English-language articles published prior to October 2016 was conducted. In addition, hand searching of bibliographies from relevant information sources was performed. Finally, solicitation of unpublished quality improvement studies was attempted by posting requests for data on both the Laboratory Medicine Best Practices website (www.cdc.gov/labbestpractices/index.html) and two ASM members-only listservs supported by the American Society for Microbiology: ClinMicroNet and ASM Division C. Further description of the search protocol as well as the full electronic search strategy for each searched database are provided in Appendix SC in the supplemental material. There was not enough literature that could be pulled during the established time frame for NAAT followed by toxin testing. This will need to be included in the update to this systematic review.

### Appraise: Screening and Evaluation of Individual Studies and Qualitative Determination of Quality and Effect

Screening of search results against eligibility criteria was performed by two sets of independent reviewers, with disagreement mediated by a third reviewer.

Studies were then abstracted and quality appraised by the volunteers using a standard data abstraction form tailored to the topic of this systematic review (see Appendix SD in the supplemental material) and further adapted for use with the Systematic Review Data Repository (SRDR) online tool (https://srdr.ahrq.gov/). The completed data abstraction forms for each included study (referred to as “evidence summary tables”) represent consensus between two independent abstractors on content and quality appraisal, with a statistician’s review of abstracted statistical data and input of qualitative effect size ratings. Use of the data abstraction forms generated the evidence summary tables for all included studies (Appendix SE).

Studies were classified as “NAAT only,” “GDH positive (GDH^+^) EIA plus NAAT,” and “GDH^+^ and toxin negative EIA plus NAAT” (Table 1) for the purposes of this systematic review.

**TABLE 1:** Assays evaluated in this systematic review

Assay (manufacturer)^*a*^
NAAT only
BD GeneOhm C diff (Becton Dickinson, Sparks, MD)
Lyra Direct C diff (Quidel, San Diego, CA)
Illumigene (Meridian Bioscience, Cincinnati, OH)
Verigene (Luminex, Austin, TX)
ProGastro C. difficile (Gen-Probe Prodesse, Waukesha, WI)
Xpert C. difficile (Cepheid, Sunnyvale, CA)
Xpert C. difficile Epi (Cepheid, Sunnyvale, CA)
Portrait toxigenic C. difficile assay (Great Basin, West Valley, UT)
AdvanSure CD RT-PCR (LG Life Sciences, South Korea)
BD Max Cdiff (Becton, Dickinson, Franklin Lakes, NJ)

GDH^+^, NAAT
C. Diff CHEK-60 EIA (GDH) (Techlab, Blacksburg, VA) → Xpert C. difficile Epi
C. Diff CHEK-60 EIA (GDH) → Xpert C. difficile
C. Diff CHEK-60 EIA (GDH) → BD GeneOhm Cdiff assay
Quick Chek GDH (Alere, Waltham, MA) → Illumigene (Meridian Bioscience, Cincinnati, OH)
C. Diff CHEK-60 EIA (GDH) → BD GeneOhm Cdiff assay
C. Diff CHEK-60 EIA (GDH) → ProGastro CD (Prodesse, Waukesha, WI)

GDH^+^, toxin negative, NAAT
C. diff Quik Chek complete (Techlab, Blacksburg, VA) → GenomEra (Abacus Diagnostica, Turku, Finland)
C. diff Quik Chek complete → Xpert C. difficile
C. Diff CHEK-60 EIA (GDH) → ProSpecT C. difficile toxin A/B (Remel/Thermo Fisher, Lenexa, KS) → BD GeneOhm Cdiff assay
C. diff Quik Chek complete → Quik Chek direct (Techlab, Blacksburg, VA) → in-house PCR of *tcdB*
C. diff Quik Chek complete → Illumigene
Premier C. difficile GDH combined with ImmunoCard → Illumigene
C. diff Quik Chek complete → Prodesse ProGastro CD
C. diff Quik Chek complete → BD GeneOhm Cdiff assay

a → indicates a subsequent test. RT-PCR, reverse transcription-PCR.

#### Quality and effect within articles.

##### (i) Study risk of bias within individual articles.

Since the primary CDC LMBP approach for evaluating study quality is not designed for assessing risk of bias (ROB) for diagnostic accuracy studies, the Quality Assessment of Diagnostic Accuracy Studies (QUADAS-2) tool was adapted for use with the LMBP method (34) (Table 2). Using QUADAS-2, two members of the expert panel independently assessed each study’s ROB, and applicability to this review’s topic, in relation to four domains: patient selection, index test, reference standard, and study flow and timing.

**TABLE 2:** Questions from QUADAS-2 used by the expert panel to evaluate studies^*a*^

Domain	Patient selection	Index test	Reference standard	Flow and timing
Description	Describe methods of patient selection; describe included patients (prior testing, presentation, intended use of index test, and setting)	Describe the index test and how it was conducted and interpreted	Describe the reference standard and how it was conducted and interpreted	Describe any patients who did not receive the index test(s) and/or reference standard or who were excluded from the 2-by-2 table^*b*^; describe the time interval and any interventions between index test(s) and reference standard
Signaling question (yes/no/unclear)	Was a consecutive or random sample of patients enrolled?	Were the index test results interpreted without knowledge of the results of the reference standard?	Is the reference standard likely to correctly classify the target condition?	Was there an appropriate interval between index test(s) and reference standard?
Risk of bias (high/low/unclear)	Was a case-control design avoided?	If a threshold was used, was it prespecified?	Were the reference standard results interpreted without knowledge of the results of the index test?	Did all patients receive a reference standard?
Concerns regarding applicability (high/low/unclear)	Did the study avoid inappropriate exclusions?	Are there concerns that the index test, its conduct, or its interpretation differed from the review question?	Are there concerns that the target condition as defined by the reference standard does not match the review question?	Did all patients receive the same reference standard?

a Adapted from reference 34 with permission of the publisher.

b See the flow diagram in reference 34.

However, the QUADAS-2 tool provides no direct means to derive an LMBP qualitative quality rating, which is based on quality point assignment and is an essential component for deriving an LMBP practice recommendation (as described below). To meet this challenge, a key adaptation of QUADAS-2 was quality point assignment as follows: analyst responses to QUADAS-2 risk-of-bias and applicability signaling questions were categorized as 1 for yes and as 0 for either unclear or no. In the absence of the capacity for analysts to discuss risk-of-bias decisions, rating disagreements among analysts on the QUADAS-2 questions were resolved in the following manner: (i) ratings for the signaling and applicability questions were coded as 0 for “no,” 0.5 for “unclear,” and 1 for “yes,” and (ii) ratings were averaged. The averaged risk-of-bias rating was then summed for each study. In applying the CDC LMBP method, scores of 8 to 10 received a quality rating of “good,” scores of 5 to 7 received a quality rating of “fair,” and scores of ≤4 received a quality rating of “poor” (34). In accord with the CDC LMBP method, studies receiving a poor quality rating are excluded from subsequent qualitative and quantitative syntheses. The list of arms by analysis is shown in Appendix SF in the supplemental material.

##### (ii) Level of effect within individual studies.

Since diagnostic accuracy studies report two related effects (sensitivity and specificity), an approach was needed to capture the trade-off between these two measures of effect as well as the clinical meaning of this trade-off. Additionally, an approach for deriving a single qualitative effect size rating from these measures was needed, as a necessary step when using the CDC LMBP method. The solution was based on two diagnostic accuracy effect measures: the positive likelihood ratio (+LR) (true-positive rate/false-positive rate) and the negative likelihood ratio (−LR) (false-negative rate/true-negative rate). Furthermore, the approach adopts cutoff points described by Deeks and Altman as providing a test’s ability to rule in or rule out a disease and extends them into the following +LR and −LR pairings (35):

- Substantial effect rating, if +LR is >10 and −LR is <0.1
- Moderate effect rating, if +LR is >10 and −LR is >0.1 or +LR is <10 and −LR is <0.1
- Minimal effect rating, if +LR is <10 and −LR is >0.1.

In other words, the cutoffs represent thresholds for “high” clinical validity, or a “high” test information value (e.g., for determinations of posttest probability of disease for individual patients), creating a basis for judging effect sizes.

The last step in deriving a single qualitative effect size rating for each study was integrating these cutoffs into a four-quadrant likelihood ratio scatterplot of positive and negative likelihood ratio pairings, as described in the next section. In summary, this approach allows for derivation of a single effect size rating for diagnostic accuracy and allows a diagnostic accuracy evidence base synthesized using the unique qualitative synthesis approach of the CDC LMBP method.

##### (iii) Overall strength (level of effect) across studies.

There are two considerations for evaluation of effect size for diagnostic accuracy statistics: (i) identifying some overall index of sample size as it relates to the interplay between sensitivity and specificity and (ii) weighing the relative risks to the patient for lower sensitivity versus lower specificity. To create an overall index of effect, the likelihood ratio scatter matrix was utilized (36).

**FIG 2:**
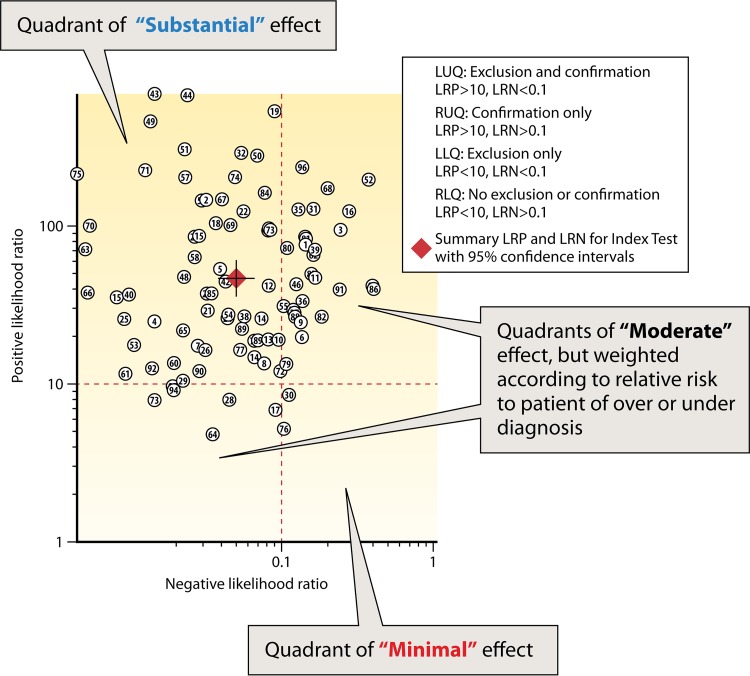
Example use of the likelihood ratio scatter matrix to aid in the decision of effect size. LUQ, left upper quadrant; RUQ, right upper quadrant; LLQ, left lower quadrant; RLQ, right lower quadrant; LRP, positive likelihood ratio; LRN, negative likelihood ratio.

Figure 2 demonstrates the likelihood ratio scatter matrix, which provides a practical tool to estimate the clinical validity of CDI testing approaches, based on where paired positive and negative likelihood ratios fall within the matrix quadrants (36). When paired likelihood ratios are within the areas that are typically used to indicate high clinical validity (+LR of >10 and −LR of <0.1), the expert panel could describe this as a “substantial” effect, especially if the error bands of the estimate (as represented by the cross hairs on the summary diamond) do not cross into other quadrants.

In terms of utility arising from the four combinations of −LR and +LR that are based on the likelihood ratio cutoffs described above, the scatter matrix quadrants can be further expressed as follows: the upper left quadrant signals a test that is good for both ruling in (confirming) and ruling out (excluding) a target condition, the upper right quadrant signals a test that is primarily good for ruling in, the lower left quadrant signals a test that is primarily good for ruling out, and the lower right quadrant signals a test that is not good at either ruling in or ruling out.

### Analyze: Data Synthesis (Meta-analysis) and Strength of the Body of Evidence

Two analytic approaches were used in this systematic review: qualitative determinations of overall strength of evidence and quantitative meta-analysis. For the qualitative analysis, studies, grouped by testing approach, were classified according to overall strength of body of evidence, with ratings of “high,” “moderate,” “suggestive,” or “insufficient.” These qualitative ratings take into account the number of studies within a group, their effect size ratings, and their quality ratings. Criteria in Table 3 are the minimum criteria to achieve a particular LMBP strength-of-evidence rating. These criteria are the basis of the body-of-evidence qualitative analyses appearing in Results below and are the primary determinant of the best practice recommendation categorizations appearing in Conclusions below.

**TABLE 3:** Criteria for determining strength of body-of-evidence ratings^*a*^

Strength of evidence	No. of studies	Effect size rating	Quality rating
High	≥3	Substantial	Good

Moderate	2	Substantial	Good
	≥3	Moderate	Good

Suggestive	1	Substantial	Good
	2	Moderate	Good
	≥3	Moderate	Fair

Insufficient	Too few	Minimal	Fair

a Adapted from reference 28 with permission of the publisher. Also see reference 33.

For the C. difficile diagnostic accuracy questions, the expert panel determined that the relative harms of false-positive and false-negative results (and, therefore, of over- or underdiagnosis) are relatively equal. Although the method used to determine the body of evidence can be adjusted to fit the demands of the specific disease and diagnostic situation (36), the work group determined that this was not necessary. Thus, no adjustments were made to the basic schema pictured in Fig. 2. Finally, this approach to determining effect size applies only to diagnostic accuracy studies with outcome measures based on rates of false-positive and false-negative results (e.g., diagnostic likelihood ratios). For nondiagnostic accuracy studies (e.g., the repeat NAAT group), the review team and expert panel utilized clinical judgment to make a determination of the effect size rating. For this group, the percent diagnostic yield was determined by the percentages listed in the specific study, representing the extent to which a suspected diagnosis (CDI) was confirmed upon repeat testing by NAAT.

#### Establishing LMBP practice recommendation categorization.

The qualitative quality ratings and qualitative effect size ratings from the individual studies for each clinical question were aggregated into bodies of evidence. The consistency of effects and patterns of effects across studies and the rating of the overall strength of the body of evidence (high, moderate, suggestive, and insufficient) were based on both qualitative and quantitative analyses using the modified LMBP process described above. Estimates of effect and the strength of the body of evidence were then used to translate results into one of three evidence-based recommendations (“recommend,” “no recommendation for or against due to insufficient evidence,” or “recommend against”). If the effect was favorable, and the overall strength of the body of evidence was either high or moderate” a practice was rated as “recommended.” When the overall strength of the body of evidence was either suggestive or insufficient, a practice was rated as “no recommendation for or against due to insufficient evidence.” Categorizations of “recommend against” are used in cases where a practice is found to be antagonistic to intended outcomes (e.g., through economic outcomes, length of stay [LOS], and delay of treatment aggregated across studies in the review).

#### Statistical analysis.

Bivariate and HSROC (hierarchal summary receiver operating characteristic) (37) models were used to estimate the summary statistics and obtain summary receiver operating characteristic (SROC) results. Analyses were carried out using the Stata 14 (Stata Corp., College Station, TX) midas command. Extreme outliers and highly influential cases were reevaluated and corrected by returning to the original article to determine if the values were accurate. The potential influences of quality criteria and preanalytic processes were evaluated via metaregression. Model diagnostics were used to evaluate the veracity of the data. Extreme outliers and highly influential cases were reevaluated and corrected as described above if appropriate. DerSimonian-Laird models were used to estimate pooled effects of proportions of patients transitioning from negative to positive for the repeat NAAT question (38).

## RESULTS

**FIG 3:**
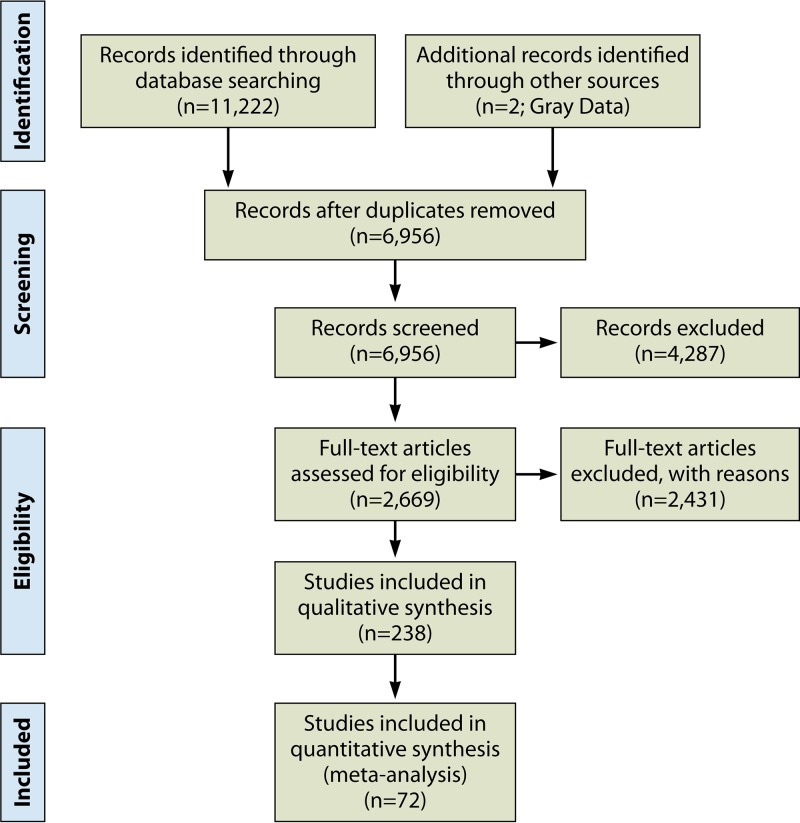
Study selection flow diagram.

A total of 11,222 bibliographic records were identified through three electronic databases. The bibliographic records included published studies as well as conference abstracts and proceedings. Seven unpublished studies were successfully obtained for screening. After removing duplicates, a total of 6,956 bibliographic records were identified. Following the elimination of duplicate papers, the respective review and technical coordinators (J. W. Snyder and C. S. Kraft) initially screened, independently, the titles and abstracts of the 6,956 studies. Of these, 4,287 studies were excluded on the basis of not having met the following defined inclusion criteria: (i) the study did not provide valid and useful information, (ii) the patient population (>18 years of age) was not defined, (iii) there was a lack of an appropriate reference standard for comparative purposes, (iv) the study failed to address the formal study questions, (v) NAAT was not included, (vi) the article was a commentary or opinion, and (vii) the practice was not sufficiently described. Screenings of titles and abstracts independently by two reviewers (C. S. Kraft and J. W. Snyder) resulted in 2,669 studies to be considered for inclusion by full-text review. Subsequent full-text screening resulted in 238 studies determined eligible for inclusion in the systematic review, eliminating 2,431 studies not meeting inclusion criteria. Of these 238 studies, 67 could be subjected to meta-analysis due to the presence of necessary information. Five studies on the topic of repeated testing by NAAT were included, bringing the total number of studies included to 72. The study selection flow diagram is depicted in Fig. 3. Full bibliographic information for each study is provided in Appendix SE in the supplemental material.

Studies that cleared this initial screening were then abstracted by two independent reviewers and evaluated for methodological quality by the expert panel (Appendix SA). For eligible studies, information on study design, study characteristics, index and reference tests, outcome measures, and findings of the study was extracted using a standardized form adapted from the LMBP data collection tool and collected into an Agency for Healthcare Research and Quality (AHRQ)-funded online data collection platform (the Systematic Review Data Repository [https://srdr.ahrq.gov/]) (Appendix SC).

### Risk of Bias within and across Studies

Results of the risk-of-bias assessment for each of the studies included in the analyses for question 1 (NAAT only) and questions 2 and 3 (NAAT-containing algorithm) are presented in Table 4, and ROB results for question 4 (repeat testing) are reported in Table 5.

**TABLE 4:**
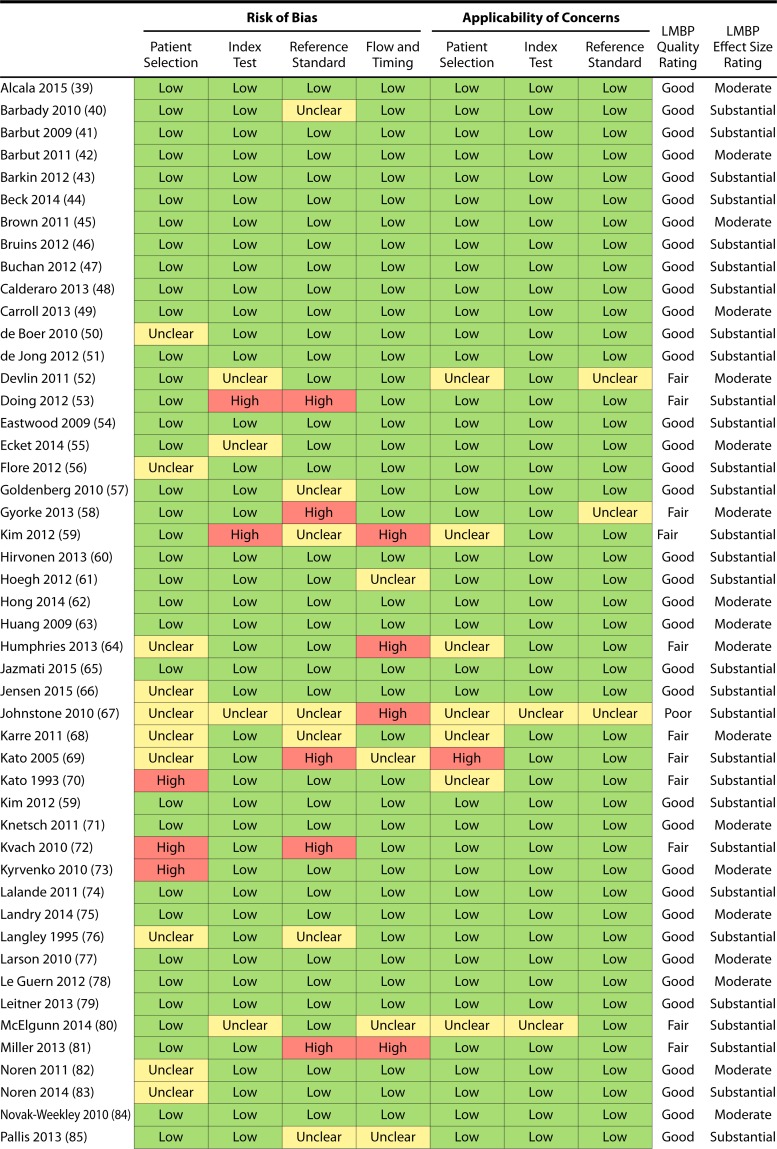

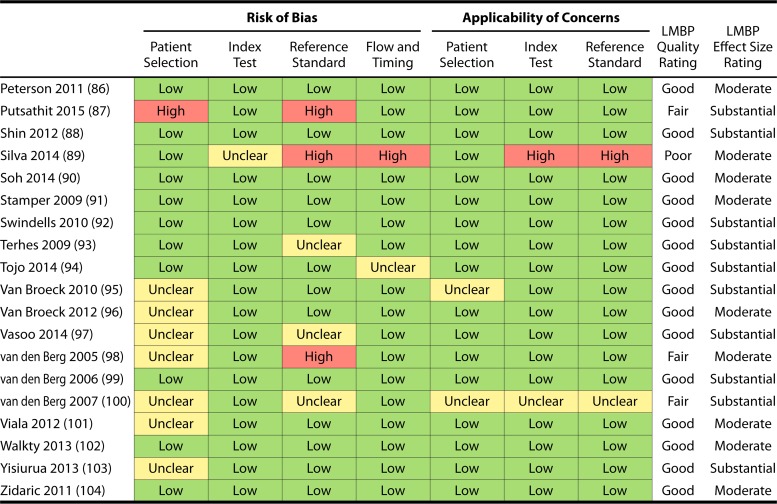
QUADAS-2 risk-of-bias results and LMBP quality and effect sizes by study^*a*^

a See references 39–104.

**TABLE 5:**
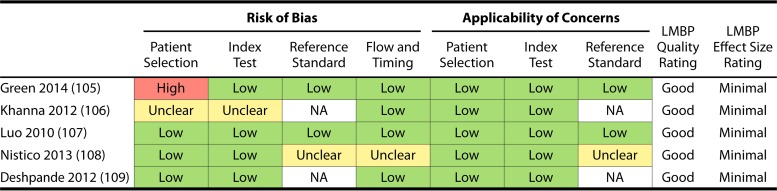
QUADAS-2 risk-of-bias results by study for studies examined for repeat testing by NAAT^*a*^

a See references 105–109. NA, not applicable.

Across studies and QUADAS-2 ROB domains included in the analysis for the first three questions, the large majority of ROB criteria were met (84.0%; 394/469). Thirty-two (47.8%) of the 67 studies had at least one ROB criterion rated as something other than a “low” risk of bias.

The two QUADAS-2 criteria in which risks were predominantly clustered were “patient selection” (31.3% of studies with some risk of bias) and “reference standard” (26.9% of studies with some risk of bias). For the patient selection criterion, the primary concern was that preanalytic procedures were not well specified. In other words, it was not made clear by the authors whether stool samples met any particular criteria (e.g., “conforms to the shape of the container”) before being included in the study, nor was it made clear whether the stool consistency was medication related, such as from laxative use. Over one-quarter of the studies (25.4%) were rated unclear for risk of bias on this criterion. Similarly, 6.0% of studies provided enough evidence to determine that the preanalytic procedures were not met (and, thus, posed a high risk of bias) for patient selection. For the repeat testing question (Table 5), patterns for risk of bias were similar (80.6% [25/31] of criteria across studies and domains with low ROB), showing a similar weakness in the patient selection criterion.

### Diagnostic Accuracy of NAAT Only and NAAT Combined with Other Tests

A total of 117 comparisons of NAAT only or NAAT-containing algorithms (GDH/NAAT or GDH/toxin/NAAT) to either TC or CCNA were extracted from 67 unique studies (Table 6). Across test arms (i.e., NAAT only, GDH/NAAT, or GDH/toxin/NAAT), the pretest probabilities (or prevalences) of the presence of C. difficile ranged from 11% to 17%, based on percent positive results with the reference standard, and therefore, predictive values should be interpreted accordingly. Of note, the number of comparisons within each arm differed dramatically, which substantially affects not only the accuracy of the estimates but also the confidence in the estimates. These results demonstrate that there is confidence in the diagnostic accuracy findings for the NAAT-only arm but less confidence in the exact estimates of the GDH/NAAT and GDH/toxin/NAAT arms, which is due to sample size.

**TABLE 6:** Diagnostic accuracy statistics by number of tests

Parameterxs^*a*^	Value for test					
	NAAT only		GDH/NAAT		GDH/toxin/NAAT	
	Estimate	95% CI	Estimate	95% CI	Estimate	95% CI
No. of studies	96		12		9	
Prevalence	0.17		0.11		0.13	
Sensitivity	0.95	0.94–0.96	0.91	0.86–0.95	0.89	0.84–0.92
ICC SEN^*b*^	0.27	0.18–0.35	0.10	0.00–0.23	0.03	0.00–0.15
Specificity	0.98	0.97–0.98	0.99	0.98–1.0	0.99	0.98–1.00
ICC SPE^*c*^	0.27	0.19–0.34	0.25	0.00–0.53	0.26	0.00–0.62
Positive likelihood ratio	46.0	35.7–59.2	113.5	49.9–258.1	155.8	57.7–420.2
Negative likelihood ratio	0.05	0.04–0.06	0.09	0.06–0.14	0.11	0.08–0.16
Diagnostic odds ratio	934	652–1,338	1,282	484–3,395	1,383	436–4,388

a ICC, interclass correlation coefficient; SEN, sensitivity; SPE, specificity.

b Proportion of total variance in sensitivity explained by between-study variation.

c Proportion of total variance in specificity explained by between-study variation.

There were three reference methods included in the analyses: TC, CCNA, and combined TC and CCNA. The breakdown of reference methods used by test algorithm is presented in Table 7, with counts representing individual studies.

**TABLE 7:** Reference method frequencies by NAAT only, GDH/NAAT, or GDH/toxin/NAAT

Reference method^*a*^	No. of studies			
	NAAT only	GDH/NAAT	GDH/toxin/NAAT	Total
TC	61	10	4	75
CCNA	26	2	5	33
Combined	10	0	0	10

Total	97	12	9	118

a TC, toxigenic culture; CCNA, cell cytotoxicity neutralization assay.

Due to the small number of studies in the GDH/NAAT and GDH/toxin/NAAT scenarios for the different reference methods, diagnostic accuracy subgroups based on these observed differences in reference methods could not be constructed for each test algorithm approach. However, Table 8 provides diagnostic accuracy statistics grouped for each of the three reference method approaches (toxigenic culture, CCNA, and both used in combination) observed in the evidence base (Table 7). This sensitivity analysis has the tests aggregated as one comparator to each reference method. The purpose of this sensitivity analysis was to determine if the diagnostic accuracies of these assays were different if they were compared to a different reference standard.

**TABLE 8:** Sensitivity analysis of diagnostic accuracy statistics by reference standard^*a*^

Parameter	Value					
	Toxigenic culture		CCNA		Combined TC/CCNA	
	Estimate	95% CI	Estimate	95% CI	Estimate	95% CI
No. of studies	74		33		10	
Prevalence	0.16		0.16		0.21	
Sensitivity	0.94	0.92, 0.95	0.93	0.93, 0.95	0.99	0.96, 1.00
ICC SEN^*b*^	0.22	0.13, 0.31	0.17	0.06, 0.28	0.39	0.03, 0.74
Specificity	0.99	0.98, 0.99	0.98	0.96, 0.98	0.98	0.96, 0.99
ICC SPE^*c*^	0.26	0.18, 0.35	0.30	0.17, 0.43	0.32	0.04, 0.60
Positive likelihood ratio	65.3	48.7, 87.8	38.5	24.9, 59.5	57.5	24.3, 135.9
Negative likelihood ratio	0.06	0.05, 0.08	0.08	0.05, 0.11	0.01	0.00, 0.04
Diagnostic odds ratio	1,079	745, 1,563	509	302, 857	5,022	1,127, 22,377

a CCNA, cell cytotoxicity neutralization assay; TC, toxigenic culture; ICC, interclass correlation coefficient.

b Proportion of total variance in sensitivity explained by between-study variation.

c Proportion of total variance in specificity explained by between-study variation.

While the specificity observed in Table 6 remained very high across arms (0.98 to 0.99), the sensitivity for detection of C. difficile decreased as additional tests were added prior to the NAAT, decreasing from 0.95 for NAAT only to 0.89 for GDH/toxin/NAAT. While all arms can be expected to be highly specific, there may be decreases in sensitivity when a GDH/toxin/NAAT algorithm is used. In general, a progressive decrease in overall diagnostic sensitivity (or overall sensitivities no greater than the lowest sensitivity of an individual component test) may be observed as one applies additional testing when the initial test results are positive within a combined testing algorithm. There is a progressive increase in overall diagnostic specificity (or overall specificities at least as high as the highest specificity of an individual component test) that may be observed as one progresses through such an algorithm (110).

The positive likelihood ratio (+LR) indicates how much more likely a person with the C. difficile organism, toxin gene, or toxin in the stool is to have a positive result on the NAAT only or NAAT algorithm than a person without the organism, toxin gene, or toxin in the stool. Typically, these ratios refer to individuals having the disease or not having the disease, but since these studies are comprised of a positive or negative test rather than predicting the disease, the LR in this review refers to presence of the organism, toxin gene, or toxin. Thus, a +LR of 46 indicates that a person with the C. difficile organism or toxin detected is 46 times more likely to have a positive result (Table 6) than a person who does not have the C. difficile organism or toxin. On the other hand, the negative likelihood ratio (−LR) indicates how less likely a person with the C. difficile organism or toxin detected is to have a negative result than a person without the organism or toxin. Thus, a −LR of 0.05 indicates that a person with the disease is 20 times less likely (1/0.05) to test negative on NAAT only (Table 6) than a person without the organism. A suggested rule of thumb for “high” information value of a diagnostic test (and, therefore, high clinical validity) is to have a +LR of ≥10 and a −LR of ≤0.1 (i.e., with a +LR of ≥10, there is a high likelihood that the disease is present when the test result is positive, while with a −LR of ≤0.1, there is a high likelihood that the disease is absent when the test result is negative) (35).

When comparing likelihood ratios across arms, all three arms have +LR in the “high” test information value range, all >10. We caution the reader against interpreting the differences across arms in +LRs as “more is better.” For the GDH/NAAT and GDH/toxin/NAAT arms (Table 6), the confidence intervals on the LRs are very broad (due, in part, to the smaller number of studies), and so there is less confidence in the point estimates of the +LRs for the GDH/NAAT and GDH/toxin/NAAT arms. It is justified to conclude that a positive C. difficile result using NAAT only or NAAT algorithms is substantially more likely in patients with the presence of the organism than in patients without the presence of C. difficile.

For −LR, the findings are less consistent. While the −LR for the NAAT only is 0.05, the LR for the GDH/NAAT is 0.09 (95% confidence interval [CI], 0.06 to 0.14), with the 95% confidence interval including the 0.1 cutoff.

This indicates that we cannot be entirely confident that the −LR point estimate for the GDH/NAAT arm actually meets the criterion for high information value (i.e., a high likelihood that the disease is absent when the test result is negative) (35). For the GDH/toxin/NAAT arm, the point estimate of the −LR falls above the high information value cutoff (−LR = 0.11 [95% CI, 0.08 to 0.16]), and the confidence interval indicates that the GDH/toxin/NAAT arm may be anywhere from 6.25 to 12.5 times less likely to return a negative result for a person with the presence of C. difficile than for a patient without the presence of C. difficile. In short, the false-negative rate for the GDH/toxin/NAAT arm appears to be higher than that for the NAAT-only arm and may even surpass the <0.1 high information value threshold. The reader is cautioned, however, against simply interpreting the GDH/toxin/NAAT algorithms as being less accurate than the NAAT only or GDH/NAAT, due to the small number of comparisons available for analysis. The most conservative interpretation would be that while we have confidence in the utility of the −LR to identify negative results in the NAAT-only arm, we have less confidence in the GDH/NAAT or GDH/toxin/NAAT arms, because of the small sample sizes.

**FIG 4:**
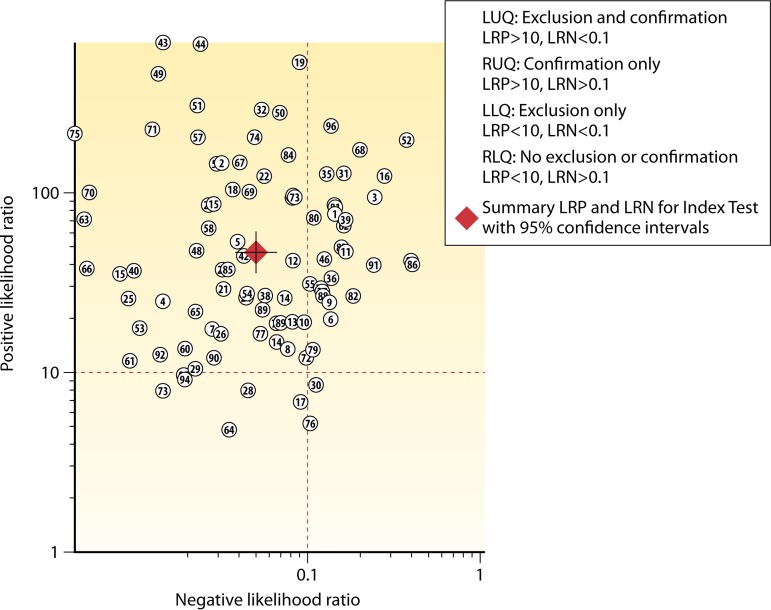
Scatter matrix of positive and negative likelihood ratios for NAAT-only detection of C. difficile. The red solid dots, in the scatter matrices, indicate the position of the combined +LR and −LR estimates. The whiskers running through the red dot are the confidence intervals for either +LR (vertical whiskers) or −LR (horizontal whiskers).

**FIG 5:**
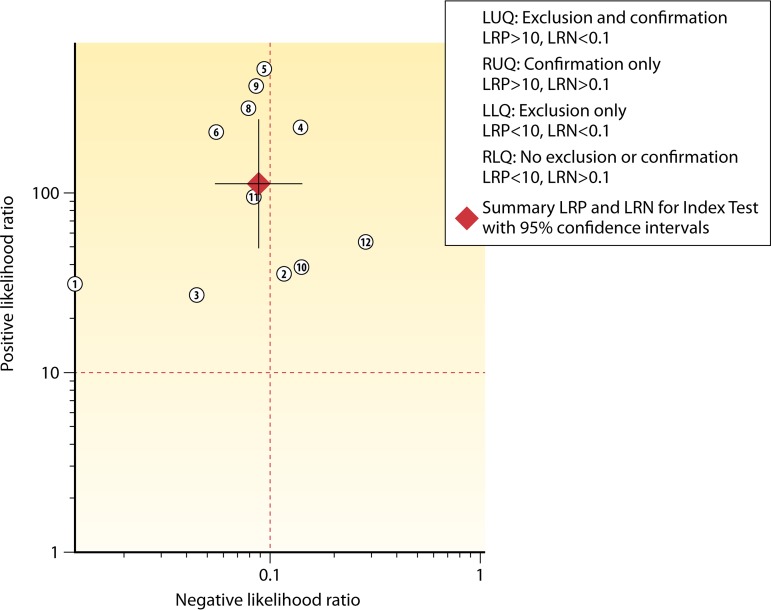
Scatter matrix of positive and negative likelihood ratios for GDH/NAAT algorithm detection of C. difficile. The red solid dots, in the scatter matrices, indicate the position of the combined +LR and −LR estimates. The whiskers running through the red dots are the confidence intervals for either +LR (vertical whiskers) or −LR (horizontal whiskers).

**FIG 6:**
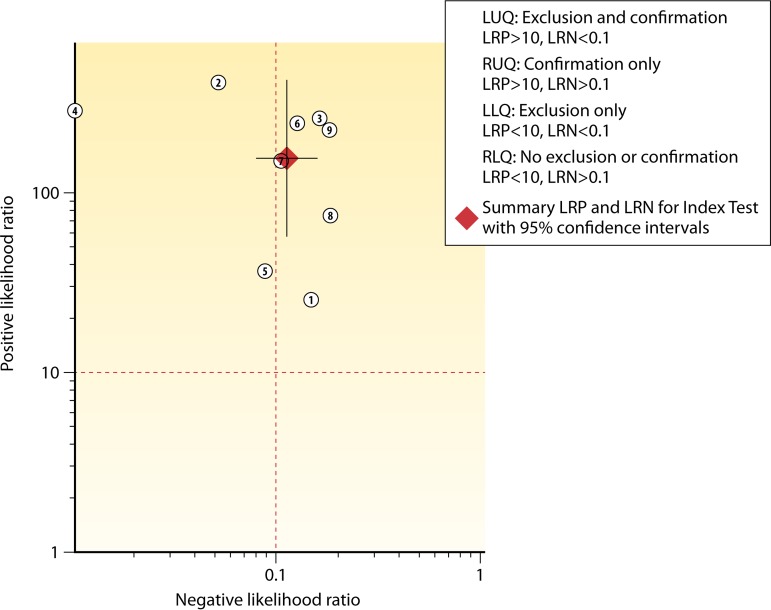
Scatter matrix of positive and negative likelihood ratios for GDH/toxin/NAAT algorithm detection of C. difficile. The red solid dots, in the scatter matrices, indicate the positions of the combined +LR and −LR estimates. The whiskers running through the red dots are the confidence intervals for either +LR (vertical whiskers) or −LR (horizontal whiskers).

The relationships between the +LR and −LR for the three arms are pictured graphically in scatter matrices (Fig. 4 to 6). The upper left quadrant of the matrices indicates the area where both +LR and −LR meet their clinical thresholds (thus, the test is useful for both excluding [i.e., accurate for true-negative results] and confirming [i.e., accurate for true-positive results] a target condition). Note that in Fig. 4 (NAAT only), not only is the summary point fully within the upper left quadrant, but the very tight confidence intervals remain within that quadrant. In contrast, while the GDH/NAAT summary point falls within the upper left quadrant (Fig. 5), the −LR confidence interval crosses into the upper right quadrant. This gives us less confidence that a GDH/NAAT diagnostic test is likely to accurately identify patients without the C. difficile organism or toxin gene. For the GDH/toxin/NAAT arm (Fig. 6), the summary point falls within the upper right quadrant, again causing us to be less confident that the GDH/toxin/NAAT solution can accurately identify patients without the C. difficile organism, toxin, or toxin gene. Study 4 in Fig. 6 is an outlier, but if excluded (data not shown), the findings would not change.

### Model Diagnostics

Since meta-analytic procedures model the summary, estimations are only as trustworthy as the models. Thus, evaluations of model diagnostics (e.g., goodness of fit) are important (111). While there were influential outliers in both the NAAT-only and GDH/NAAT arms, these outliers were likely to pull the estimates in Table 6 slightly lower. Hence, the estimates for both the NAAT-only and the GDH/NAAT arms presented in Table 6 may be slightly conservative. However, in the GDH/toxin/NAAT arm, there was a single outlier (one study) that biased the model estimates upward, likely resulting in an overestimate of the diagnostic accuracy statistics presented in Table 6. We attempted to carry out a sensitivity analysis on the results of the GDH/toxin/NAAT arm by removing the influential case and then recomputing the statistics. However, because of the small number of comparisons, the revised model failed to achieve convergence, and no solution could be computed. Thus, readers are cautioned that while the NAAT-only and the GDH/NAAT arm estimates may be somewhat conservative (i.e., the sensitivity and specificity may be slightly higher than reported), the values for the GDH/toxin/NAAT arm may overestimate the diagnostic accuracy in this arm. Readers are encouraged to perform and contribute additional study data to add to the data available in order to refine future analyses (see Appendix SB in the supplemental material for study components needed).

### Heterogeneity

Since there are variations in sample characteristics and preanalytic and analytic procedures, heterogeneity can be assumed in diagnostic accuracy meta-analyses. However, unlike meta-analyses of treatment studies, there are no generally accepted measures of heterogeneity for diagnostic accuracy meta-analyses (112). The interclass correlation coefficients (ICCs) reported in Table 6 indicate that approximately one-quarter of the differences in study findings are due to between-study differences. The analyses of heterogeneity described below are intended to explain some of the differences between studies. Two possible sources of variation were examined to determine the source of the heterogeneity and examine why some studies find higher levels of diagnostic accuracy than others. These two possible sources of variation were (i) the NAAT used and (ii) the preanalytic procedure of ensuring that the sample was unformed (i.e., conformed to the container).

#### Device as a cause of heterogeneity.

Due to the limited number of studies available in the GDH/NAAT and GDH/toxin/NAAT arms, and because these comparisons would also introduce variation from the companion devices (e.g., GDH and toxin), we descriptively compared device (platform) differences only within the NAAT-only arm (Table 9). While there are some differences among devices, all +LR point estimates were >10, and all −LR point estimates were <0.1 (35). Only the 95% confidence interval for the GeneOhm device contained the clinical cutoff threshold for the −LR. Due to variations in other study characteristics (e.g., differences in samples or differences in analytic or preanalytic procedures), we are hesitant to conclude that one device performs better or worse than other devices. Rather, all devices appear to perform roughly the same. Thus, although variations in NAAT devices may have contributed to some variation in findings across studies, the statistics presented in Table 9 demonstrate that the variation is likely to be relatively minor.

**TABLE 9:** NAAT-only device comparison

Device and parameter	Value	
	Estimate	95% CI
GeneOhm (*n* = 20)		
Sensitivity	0.92	0.88–0.94
Specificity	0.98	0.97–0.99
Positive likelihood ratio	48.4	30.0–78.0
Negative likelihood ratio	0.09	0.06–0.12
Diagnostic odds ratio	569	325–996

Illumigene (*n* = 18)		
Sensitivity	0.95	0.93–0.97
Specificity	0.99	0.98–1.00
Positive likelihood ratio	89	40.1–197.7
Negative likelihood ratio	0.05	0.03–0.07
Diagnostic odds ratio	1,909	755–4,822

In-house (*n* = 12)		
Sensitivity	0.96	0.92–0.98
Specificity	0.96	0.94–0.98
Positive likelihood ratio	26.4	15.3–45.4
Negative likelihood ratio	0.04	0.02–0.09
Diagnostic odds ratio	616	233–1,630

Xpert (*n* = 14)		
Sensitivity	0.99	0.95–1.00
Specificity	0.97	0.94–0.98
Positive likelihood ratio	30.6	17.8–52.5
Negative likelihood ratio	0.01	0.00–0.05
Diagnostic odds ratio	3,400	611–18,920

Other device (*n* = 33)		
Sensitivity	0.95	0.91–0.97
Specificity	0.98	0.97–0.99
Positive likelihood ratio	47.6	30.9–73.1
Negative likelihood ratio	0.06	0.03–0.09
Diagnostic odds ratio	854	442–1,652

#### Preanalytic procedure as a cause of heterogeneity: stool conforms to the container.

The studies were assessed for whether the consistency of stool was considered in their analysis and study design. Typically, when the preanalytical aspects were involved with sample selection, only those samples that were considered to conform to the shape of the container (e.g., diarrheal, etc.) were used in the study. Analysts were directed to extract data from each of the articles on whether the authors required that specimens “conformed to the shape of the container.” Analysts could answer “yes” (authors provide a statement confirming that this preanalytic condition was met), “no” (authors give some indication that the preanalytic condition was not met), or “uncertain” (authors do not provide any information on whether the stool conformed to the container). These categories were recoded into “yes” and “no” (combining “no” and “uncertain”). For the NAAT-only and the GDH/toxin/NAAT arms, only 50% of the comparisons confirmed that the stool conformed to the container, and only one-third of the GDH/toxin cases did so (Table 10).

**TABLE 10:** Comparison of sensitivities and specificities by whether authors reported that the stool conforms to the container^*a*^

Categorization of whether stool meets criteria reported	No. of studies in arm	Sensitivity		*P* value for sensitivity	Specificity		*P* value for specificity
		Estimate	95% CI		Estimate	95% CI	
NAAT only							
Yes	48	0.94	0.92–0.96	<0.001	0.97	0.96–0.98	<0.001
No	49	0.96	0.94–0.97		0.99	0.98–0.99	

GDH/NAAT							
Yes	7	0.91	0.86–0.96	0.02	0.99	0.98–1.00	0.16
No	5	0.92	0.86–0.98		0.99	0.98–1.00	

GDH/toxin/NAAT							
Yes	4	0.86	0.79–0.92	<0.001	1.00	0.99–1.00	0.58
No	5	0.89	0.85–0.93		0.99	0.98–1.00	

a In those studies where the stool had to meet the criteria before being tested, only the samples that met the preanalytic requirement were tested.

In studies where the authors explicitly indicated that the stool conformed to the shape of the container and tested only those that did or, its equivalent, “liquid stools” and diarrhea, etc., the diagnostic sensitivity was significantly lower than for studies where the authors did not report that the stool conformed to the shape of the container. The results were mixed for diagnostic specificity. While specificity was significantly lower in studies that reported that the stool conformed to the shape of the container in the NAAT-only arm (*P* < 0.001), there was no significant difference (*P* = 0.58) for the GDH/toxin/NAAT arm, undoubtedly due to the small number of cases in this arm. In the GDH/NAAT arm, the difference showed a trend toward significance (*P* = 0.16).

In summary, the results in Table 10 indicate that the single preanalytic practice of ensuring that the stool specimen conformed to the shape of the container contributed statistically significantly to differences in sensitivity and specificity across studies. In most cases, these diagnostic accuracy estimates are higher when authors do not confirm the presence of this preanalytic practice, which is consistent with the idea that NAAT can detect C. difficile colonization in formed stool. Essentially, the sensitivity appears higher due to the fact that the test is detecting more positive samples (which would be considered colonization) in addition to the presence of toxigenic C. difficile in a diarrheal stool sample.

### Meta-analysis of NAAT Used for Repeat Testing

Five studies that examined the increased diagnostic yield of NAAT used for repeat testing were available (Table 5). In this group, percent diagnostic yield represents the extent to which a suspected diagnosis (CDI) was made upon repeat testing using NAAT (an initial negative test followed by a positive test). The studies varied primarily in the length of time between repeat testing, with four studies reporting diagnostic yield over a 7-day period (105–107, 109), two studies reporting over a 14-day period (106, 107), and one study reporting over a 59-day period (108). All studies were retrospective in design. The number of repeat tests on negative samples was not reported in all studies, but for those that did report (106–108), tests were repeated between one and five times.

For the 7-day window for repeat testing by NAAT, the pooled proportion of subjects transitioning from negative to positive C. difficile results was 2% (95% CI, 0.009 to 0.032; *P* < 0.001). Heterogeneity was high (*I*^2^ = 85.23%; *P* < 0.001). Individual study results varied from a low of 1% transitioning (105) to a high of 3.3% (109).

For the >7-day window (14 days to 59 days), the proportion of subjects transitioning from negative to positive C. difficile results was 3% (95% CI, 0.023 to 0.038; *P* < 0.001). Heterogeneity was low (*I*^2^ = 0%; *P* = 0.482). Individual study results varied from a low of 2.1% transitioning (108) to a high of 3.4% (106).

Even though heterogeneity was high for the 7-day window, the small number of studies available prevented examination of the sources of this heterogeneity. However, given that the ranges of transition values were very narrow across studies and time periods (1% to 3.3%), there is confidence that repeat testing by NAAT testing within 7 days is unlikely to provide a substantial increase in the number of positive C. difficile results.

### Level of Evidence across Questions

Table 11 depicts the LMBP strength of body of evidence for each testing practice assessed. It represents the final level of LMBP qualitative synthesis and is based on the LMBP criteria presented in Table 3 applied to the study-level information summarized in Tables 4 and 5 as well as in Fig. 4 to 6.

**TABLE 11:** LMBP strength of body of evidence for all questions

Question	No. of studies	No. of comparisons	Effect	Quality
NAAT only, high strength of body of evidence	60	96	Substantial	Good
GDH/NAAT, high strength of body of evidence	9	12	Substantial	Good
GDH/toxin/NAAT, moderate strength of body of evidence	7	9	Moderate	Good
Repeat testing using NAAT, insufficient strength of body of evidence	5	6	Minimal	Good

While some risks of bias were identified (specifically within “patient selection” and “reference standard” criteria), the assessment of the expert panel was that these did not pose a serious threat to our confidence in the findings. In sum, the decision of the expert panel was that the quality of the evidence for all questions was good. Additionally, based on evaluations of the likelihood ratio scatter matrices (Fig. 4 to 6), the effect sizes were determined to be substantial for NAAT only, substantial for the GDH/NAAT algorithm (although uncertainty remains due to the wide confidence intervals), and moderate for the GDH/toxin/NAAT algorithm (Table 11). For repeat testing using NAAT, the effect size was minimal.

## ADDITIONAL CONSIDERATIONS

### Applicability and Generalizability

C. difficile testing utilizing NAAT-based algorithms provides diagnostically accurate detection of the C. difficile organism compared to the reference standard. C. difficile testing by NAAT should also not be repeated to increase diagnostic sensitivity after an initial negative test, since it is already a diagnostically sensitive test.

In addition, in Table 10, it is demonstrated that there was a statistically significantly decreased sensitivity when a stool criterion (such as diarrhea or unformed stool) was included in the studies. This implies that preanalytic variables affect the diagnostic accuracy of C. difficile testing. This systematic review was unable to answer the question about which diagnostic test is most accurate to make the diagnosis of C. difficile infection due to the fact that the large majority of studies do not include clinical outcomes. Therefore, this systematic review was focused on an intermediate outcome (Fig. 1) when NAAT is part of laboratory testing.

These findings indicate the need for improvement in reporting C. difficile diagnostic accuracy study results rather than genuine flaws in the research. Incomplete reporting in these peer-reviewed articles can affect the usefulness of systematic review findings. While information that is relevant to a particular diagnostic accuracy evidence base can vary (e.g., preanalytic testing criteria), the Standards for Reporting of Diagnostic Accuracy Studies (STARD) provides a list of minimum essential reporting items (113). Currently, the STARD includes a requirement for the description of eligible participants but does not specifically discuss preanalytic considerations for the samples *per se*. The influence of preanalytic factors on test performance may be better established through fuller reporting of these factors in the primary evidence base and may be considered useful for augmentation of STARD reporting standards or for ensuring that the specific preanalytical considerations fall under eligible participants.

It was determined by the expert panel that while concerns remain about how well studies report patient selection criteria, this was unlikely to compromise confidence in the results. The sensitivity analysis (Table 8) demonstrated that while the reference standards of TC and CCNA are different tests, both can be used as a reference standard without concern of decreasing diagnostic accuracy. However, studies were excluded if neither of these reference standards was used or if they were used only on discordant results. Not all studies defined the reference standard as either TC or CCNA (or both) but rather combined the outcomes of these reference standards along with other diagnostic tests to create a panel of tests to serve as the reference standard. The judgment of the expert analysts was that while the use of a panel of tests as a reference method rather than TC or CCNA alone would affect diagnostic accuracy measures, these panels were unlikely to be less accurate, and so these studies could be included in the analysis and did not compromise our confidence in the findings. However, for a study to be included in this analysis, at least TC or CCNA had to be in the panel of tests and not used only for analysis of discrepant results.

Additionally, the current evidence base on the effectiveness of C. difficile algorithms did not permit direct assessment of health benefits, whether direct health outcomes or surrogate outcomes as specified in the analytic framework (e.g., delay to treatment or delay to isolation) and other outcomes deemed relevant (e.g., LOS or intensive care unit [ICU] stay). Therefore, the clinical utility (i.e., the degree to which the use of a test is associated with improved health outcomes) of the algorithms examined remains unclear, although treatment and clinical care options resulting from the test information are well characterized.

### Comparison to Recent Clinical Guidelines

The recent IDSA/SHEA guidelines (11) outline their recommendations for practice and are based on use of Grading of Recommendations Assessment, Development, and Evaluation (GRADE) criteria. GRADE criteria differ from LMBP criteria in their assessment of an evidence base to derive practice recommendations. GRADE criteria drive a more direct accounting of the clinical factors related to C. difficile testing, and as was shown in the IDSA/SHEA guidelines, there were few studies in the literature that provided clinical outcome data. Therefore, the IDSA/SHEA guidelines report limited evidence to support testing practices because the data themselves are limited. However, the guidelines elegantly interpret their findings in the setting of the clinical decision-making process and guide their recommendations based on the clinical context (11).

In this ASM-led systematic review, practice recommendations relate to detection of the toxin or toxin gene of the organism, with a focus on relevant diagnostic accuracy measures rather than the diagnosis of CDI, which (as discussed above) is based on a combination of clinical presentation and laboratory testing. Therefore, in following the LMBP framework, this systematic review sought to assess diagnostic accuracy (an intermediate outcome in health care) as the review’s primary outcome of interest. In other words, this systematic review did not downgrade such evidence for being indirect to patient outcomes. While this review focused on identification of the cell wall enzyme, toxin, or toxin gene of the organism as the basis of guidance on diagnostic testing strategies, it is clear that diagnostic testing directly supports decisions about whether or not to treat. Furthermore, it is recommended that the preanalytic aspects of patient presentation should be taken into account with the interpretation of the test result.

### Feasibility of Implementation

Since many of the studies do not include preanalytic variables, and given the results of Table 10, testing of formed stool may lead to overdiagnosis with a sensitive test. Clinicians should utilize clinically agreed-upon symptoms, appropriate diarrheal history (at least 3 unformed bowel movements within 24 h), and antimicrobial use history as well as exclude patients on laxatives and promotility drugs (114). Prior to changing algorithms, laboratories should base their testing decision on published data, and collect and analyze the data at their institution, in order to support the new testing practices. Assays that have high sensitivity can be utilized as long as clinicians ordering the test understand the limitations for a patient who does not meet the testing criteria (11).

As emphasized in the IDSA/SHEA guidelines (11), the aspect of health care provider education on the use and interpretation of laboratory testing needs to be critically placed in the clinical context of the patient. There are initiatives that have been implemented to assist health care providers by creating criteria by which to appropriately order laboratory testing for C. difficile. Some health care systems have embarked on the use of a form in the electronic medical record that the provider must fill out in order for the test to be performed, based on certain preanalytic requirements, such as frequency and consistency of bowel movements. Empowerment of the health care workers who are collecting the stool from the patient to have discussions with clinicians about whether the stool is formed or diarrheal after visualization is also critical. In their study, Truong et al. restricted the use of C. difficile NAAT with the following requirements for orders: ≥3 unformed bowel movements over 24 h and no laxative intake during the previous 48 h. However, exceptions were made for patients admitted within the previous 24 h, for patients with a rectal or ostomy tube, and if the ordering provider called to override the rejection. This policy resulted in a significant reduction in test utilization as well as reduced oral vancomycin use in these patients (115). Truong et al. also looked at outcomes for individuals with cancelled C. difficile orders and found that they were not worse than those for individuals whose specimens were accepted and were C. difficile negative (115). Quan et al. utilized automated verification at the time of computerized provider order entry to enforce appropriate CDI testing. The criteria included (i) diarrhea (≥3 liquid/watery stools in 24 h), (ii) no reasonable alternate cause for diarrhea, (iii) no laxative use within 24 h, (iv) no previous CDI test result within 7 days, and (v) age of >1 year. This criterion-based testing protocol reduced testing by two-thirds and decreased rates of C. difficile without changing the methodology of testing (114). In addition to the published data about the policies, “gray” (unpublished) data were obtained from institutions during the call for unpublished data for this systematic review (28). Some hospitals differentiated recommendations for individuals who were in the hospital for less than or more than 4 days, and if the stay was <4 days, the individual would be tested if they had unexplained loose/unformed stools. If the stay was >4 days, the algorithm included ≥3 liquid/watery stools in 24 h, discontinuation of laxatives, and clinical signs/symptoms of C. difficile infection or epidemiological risk factors for C. difficile. There are numerous examples in the literature which have demonstrated that improved education and adherence to preanalytic criteria prior to testing lead to appropriate clinical utility despite the C. difficile test that is used.

### Limitations

A major limitation of this systematic review is that a main NAAT algorithm (NAAT followed by toxin testing) is not included due to the time scope of this study. This will be included in the future update of this systematic review. One limitation is the new use of the likelihood ratio within the LMBP method, and QUADAS-2. A justification for these methods has been published (36). A significant limitation of the evidence base was the failure to incorporate preanalytic parameters and clinical outcomes in the study design. This was very common in all of the literature that was evaluated and speaks to the fact that there should be increased emphasis placed on the context of laboratory testing as well as its diagnostic accuracy. This is especially clear in the IDSA/SHEA guidelines, where a small number of articles met criteria to be referenced for diagnostic testing recommendations (11). Given this limitation, there is currently little evidence base to assess the impact of overall testing practices on population health outcomes despite a high number of studies regarding testing for C. difficile. Reporting for health care facility onset does take into account the type of testing that is used by the facilities, so that comparisons for testing prevalence are consistent. In addition, some molecular stool testing also includes the sample being placed in liquid medium, and this will limit the ability to assess whether the stool does not conform to the shape of the container. For these types of assays, it will be difficult to determine if the preanalytical characteristics (eligibility of the sample) have been met, leading to further confusion as to what the result means for the specific patient.

## FUTURE RESEARCH

After the evaluation of a large number of studies in this systematic review, the paucity of clinical data that are collected during diagnostic accuracy studies was striking. In general, the diagnostic laboratory community should consider, in addition to diagnostic comparison studies, the inclusion of preanalytic factors and postanalytic clinical outcomes in these studies. It may be also unclear as to whether the reference standard is indeed a good representation of active toxigenic infection in a patient. If an isolate of C. difficile recovered from stool produces toxin in an *in vitro* test, such as growing the organism in an enrichment broth, it does not tell us that toxin is being produced in the patient. The presence of the organism does not tell us about its *in vivo* activity. The dilemma that has been created by the development of NAAT as a detection method pulls us away from the *in vivo* toxin activity by just detecting the presence of the organism toxin gene in the stool. There are ultrasensitive toxin tests (116) that are being developed to be able to increase the sensitivity of toxin testing without the oversensitivity of the detection of the toxin gene in a NAAT. Due to financial pressure being placed on hospitals which have high C. difficile infection rates by federal pay-for-performance programs, there are clinical microbiology laboratories that are considering changing from NAAT-only or NAAT-containing algorithms to toxin tests alone (14). These tests have been shown to be less sensitive than the three testing strategies recommended here (14). The use of insensitive toxin tests may result in an increased number of missed diagnoses, leading to poorer clinical outcomes; however, studies regarding these outcomes need to be performed in order to determine if there are poorer clinical outcomes. Outcome studies in which different testing approaches are compared are essential to determine optimal testing strategies (see Appendix SB in the supplemental material). Additional studies are needed on the yield and duration of repeat testing using NAAT-containing testing strategies. The overlay of clinical outcome on studies of repeat testing will be important for health care facility policies for acceptance of patient samples at certain time intervals.

## CONCLUSIONS

### Practice Recommendations

Recommendations are categorized as “recommended,” “not recommended,” and “no recommendation for or against due to insufficient evidence.” Recommendation categorization in this review is a function of the currently available evidence base and of the CDC LMBP method, including *a priori* analysis criteria (e.g., selected effect measure rating cutoffs, the LMBP quality assessment tool, and the LMBP strength of body of evidence matrix). The approach for recommendation categorization is described in Methods above, with criteria indicated in Table 3. ASM recommendations arising from this systematic review do not serve to endorse specific NAATs; rather, they relate to the ability to choose for each individual health care system the most appropriate C. difficile laboratory diagnostic test algorithm that best supports the practices of the institution (11).

Practice recommendations are summarized in Table 12, with additional details provided in the remainder of this section.

**TABLE 12:** Summary of ASM practice recommendations for C. difficile testing

Practice category	Practice recommendation
NAAT only	Use of NAAT-only testing is recommended as a best practice for the detection of the C. difficile toxin gene
GDH/NAAT algorithm	Use of a GDH/NAAT algorithm is recommended as a best practice for the detection of the C. difficile organism/toxin gene
GDH/toxin/NAAT algorithm	Use of a GDH/toxin/NAAT algorithm is recommended as a best practice for the detection of the C. difficile organism, toxin, or toxin gene
Repeated testing using NAAT	A recommendation for or against repeated testing for C. difficile using a NAAT as a best practice cannot be made due to insufficient evidence

### ASM Recommendation for NAAT-Only Testing

Among patients suspected of having *Clostridioides* (*Clostridium*) *difficile* infection, NAAT-only testing is a recommended practice for detection of the C. difficile toxin gene. The overall strength of evidence for this practice is rated as high. The pooled effect rating for 46 studies meta-analyzed is substantial (+LR = 46.0 [95% CI, 35.7, 59.2]; −LR = 0.05 [95% CI, 0.04, 0.06]). Effects across studies were consistent.

### ASM Recommendation for the GDH/NAAT Algorithm

Among patients suspected of having *Clostridioides* (*Clostridium*) *difficile* infection, a GDH/NAAT algorithm is a recommended practice for detection of the C. difficile organism/toxin gene. The overall strength of evidence for this practice is rated as high. The pooled effect rating for 11 studies meta-analyzed is substantial (+LR = 113.5 [95% CI, 49.9, 258.1]; −LR = 0.09 [95% CI, 0.06, 0.14]). Effects across studies were consistent.

### ASM Recommendation for the GDH/toxin/NAAT Algorithm

Among patients suspected of having *Clostridioides* (*Clostridium*) *difficile* infection, an algorithm including NAAT is a recommended practice for detection of the C. difficile organism/toxin/toxin gene. The overall strength of evidence for this practice is rated as moderate (more than 3 studies with good to moderate quality-to-effect pairings achieved a “moderate” strength-of-evidence rating). The pooled effect rating for 11 studies meta-analyzed is moderate (+LR = 155.8 [95% CI, 57.7, 420.2]; −LR = 0.11 [95% CI, 0.08, 0.16]). Effects across studies were consistent.

### ASM Recommendation for Repeated Testing Using NAAT

Among patients suspected of having *Clostridioides* (*Clostridium*) *difficile* infection, due to insufficient evidence, there is no recommendation for or against repeated testing by NAAT only within 7 days when the result is negative. The overall strength of evidence for this practice is rated as insufficient. The pooled effect rating for 5 studies meta-analyzed is minimal (3% conversion from negative to positive [95% CI, 0.023 to 0.038]). Effects across studies were consistent. A limited but consistent body of generally high-quality evidence indicates that repeat testing using NAAT has minimal additional benefit for detecting the presence of the C. difficile toxin gene.

However, while the use of the LMBP strength of body of evidence criteria sustains an “insufficient” strength-of-evidence categorization, a “minimal” effect for diagnostic yield should be interpreted to mean that repeat testing using NAAT does not appreciably contribute to patient diagnosis of CDI. Therefore, in this context, a minimal-effect finding (when combined with a good-quality evidence base) may also be interpreted as strong evidence against the use of repeat testing by NAAT. In using the LMBP method, however, a category of “recommendation against” may be achievable when outcomes for repeat testing include outcomes such as cost of testing and time to treatment, etc. In short, repeat testing by NAAT is likely a practice to be recommended against, a finding which may be more definitively sustained by future studies. See Appendix SD in the supplemental material for guidance for future studies.

## APPENDIX

### GLOSSARY

analytical sensitivity: The ability of a method to detect a low concentration of an analyte.
clinical outcomes: Measurable changes in health, function, or quality of life that result from clinical/medical care.
clinical validity: The accuracy of detection of the presence or absence of a phenotype/disease.
diagnostic odds ratio: The odds of a positive test result for those with disease relative to the odds of a positive test result for those without the disease, as can be mathematically represented by dividing the positive likelihood ratio by the negative likelihood ratio. It provides a global measure of a test’s diagnostic accuracy and can assist in comparison of diagnostic accuracies between two or more index tests. However, as an overall measure of diagnostic accuracy, it obscures the clinically important trade-off between rates of false-positive and false-negative results.
diagnostic sensitivity: The proportion of individuals correctly classified by the index test as having a disease, target condition, or gene of the infectious agent of interest.
diagnostic specificity: The proportion of individuals correctly classified by the index test as not having a disease, target condition, or gene of the infectious agent of interest.
effect size: The association between two or more studies’ outcome measures for the group in which the intervention/practice was evaluated and those for its control or comparison group. Effect size ratings can be numeric or reflect the magnitude of effect in qualitative terms. Numeric representation can be converted to qualitative values through expert consensus on cutoffs representing a “substantial,” “moderate,” or “minimal” effect; however, setting cutoffs for qualitative ratings invariably has an element of subjective judgment.
evidence summary tables: Used in systematic reviews to summarize the study findings, including but not limited to study design, author, statistical summary, quality of study, magnitude of benefit, absolute risk reduction, and number needed to treat. The content of the tables depends on the topic of the review.
*I*^2^: Statistic describing the percentage of variation across studies that is due to heterogeneity rather than chance.
interclass correlation coefficient: Measures a relation between two variables of different classes, in this case between different studies.
likelihood ratio scatter matrix: Used in this context to plot positive and negative likelihood ratio pairings for diagnostic test accuracy studies. The pairings are then graphed on one of four quadrants derived from established thresholds for test clinical validity.
meta-analysis: The process of using statistical methods to standardize and quantitatively combine the results of similar studies in an attempt to allow inferences to be made from a collection of studies. It allows for estimates of effects across studies.
preanalytical: The testing phase that occurs first in the laboratory process and may include specimen handling issues that occur even prior to the time when the specimen is received in the laboratory.
quality improvement: A systematic, formal approach to the analysis of practice performance and efforts to improve performance.
reference standard/method: The test, combination of tests, or procedure that is considered the best available method of categorizing participants in a study of diagnostic test accuracy as having or not having a target condition.
systematic review: A scientific investigation that focuses on a specific question and uses explicit, planned scientific methods to identify, select, assess, and summarize the findings of similar but separate studies. It may or may not include a quantitative synthesis of the results from separate studies (meta-analysis).
testing algorithm: Diagnostic testing that uses multiple tests in a sequence of specified actions.
test clinical utility: The extent to which a test is usefully informative by contributing to improved clinical management or patient-related outcomes. Demonstrations of a test’s utility have been achieved through randomized controlled trials of test-and-treat (or test-and-clinical response) interventions and through decision analysis modeling in which the probabilities of relevant outcomes are estimated.
